# Study of an SSA-BP Neural Network-Based Strength Prediction Model for Slag–Cement-Stabilized Soil

**DOI:** 10.3390/ma18153520

**Published:** 2025-07-27

**Authors:** Bei Zhang, Xingyu Tao, Han Zhang, Jun Yu

**Affiliations:** 1 School of Civil Engineering, Nantong Vocational University, Nantong 226007, China; 2801035@mail.ntvu.edu.cn; 2 School of Transportation and Civil Engineering, Nantong University, Nantong 226001, China; zh_970902@126.com (H.Z.); yujunhjsl@foxmail.com (J.Y.)

**Keywords:** cement-stabilized soil, SSA-BP neural network, sparrow search algorithm, prediction of compressive strength, prediction model

## Abstract

As an industrial waste, slag powder can be processed and incorporated into cement-based materials as an additive, significantly improving the engineering properties of cement–soil. The strength of slag–cement-stabilized soil is subject to nonlinear interactions among multiple factors, including cement content, slag powder dosage, curing age, and moisture content, forming a complex influence mechanism. To achieve accurate strength prediction and mix proportion optimization for slag–cement-stabilized soil, this study prepared cement-stabilized soil specimens with different slag powder contents using typical sandy soil and clay from the Nantong region, and obtained sample data through unconfined compressive strength tests. A Back Propagation (BP) neural network prediction model was also established. Addressing the limitations of traditional BP neural networks in prediction accuracy caused by random initial weight thresholds and susceptibility to local optima, the sparrow search algorithm (SSA) was introduced to optimize initial network parameters, constructing an SSA-BP model that effectively enhances convergence speed and generalization capability. Research results demonstrated that the SSA-BP model reduced prediction error by 53.4% compared with the traditional BP model, showing superior prediction accuracy and effective characterization of multifactor nonlinear relationships. This study provides theoretical support and an efficient prediction tool for industrial waste recycling and environmentally friendly solidified soil engineering design.

## 1. Introduction

As an artificial composite material, slag powder–cement-stabilized soil enhances its mechanical properties by modifying the soil’s microstructure through the cement hydration reaction [1,2]. It combines economic efficiency with construction convenience and has become the core material in foundation treatment projects [3,4,5]. Compressive strength, as a key performance indicator, is affected by the coupling of multiple parameters, such as soil properties [6], slag powder content [7], cement content [8], water–cement ratio [9], and curing age [10]. Cao’s research [11] shows that within the range of typical engineering parameters, the strength has an exponential non-linear relationship with cement content and age. Meanwhile, it is controlled by implicit variables associated with porosity, such as water content, compactness, and the amount of hydration products generated. Traditional empirical formulas are difficult to use to effectively characterize the synergistic mechanism of multiple factors. This limitation necessitates a large number of trial mix tests in engineering practice, leading to significant resource waste. Consequently, to reduce the waste of human and material resources associated with pre-construction trial mixing and post-project testing, establishing a regional multi-factor strength prediction model is urgently needed [12,13].

Most existing prediction models are limited to single or a few factors. For instance, Zhou developed separate compressive strength prediction models for cement-stabilized soil in seasonal frozen regions: one based solely on curing age [14] and another incorporating both cement mixing ratio and age. Similarly, Zhu derived relationships between compressive strength and curing age at fixed cement contents (8% and 13%) through laboratory mechanical tests [15]. With the development of artificial intelligence technology, neural networks offer a new approach to multidimensional modeling, thanks to their powerful nonlinear mapping capabilities. Liu’s ANN-based model [16] for cement-reinforced soil mechanical properties exemplifies this, demonstrating efficacy in resolving complex nonlinear problems. However, traditional ANNs exhibit drawbacks such as high sample dependency and hyperparameter sensitivity, limiting their adaptability to engineering contexts with scarce or fluctuating data. In contrast, BP neural networks dynamically adjust the network weights through the backpropagation algorithm. They integrate robust nonlinear fitting with self-learning capabilities, effectively addressing pattern recognition, function approximation, and prediction in complex systems—particularly excelling in multivariate nonlinear modeling [17,18,19,20]. However, the performance of the BP neural network highly depends on the setting of the initial weights and thresholds. Improper initialization may lead to low efficiency in searching the parameter space: excessively large initial values are likely to cause gradient explosion, resulting in a nonlinear increase in prediction errors, while excessively small initial values may cause gradient disappearance, causing the model to fall into local optima and overfit [21]. This vulnerability stems from the error backpropagation mechanism’s dependence on the initial parameter space configuration. Consequently, pretraining weights and thresholds with intelligent optimization algorithms have emerged as a pivotal strategy for overcoming the convergence limitations of conventional BP networks [22,23,24].

In this study, mineral powder cement soil specimens with different mix ratios were prepared using the typical silty sand and silty clay in the Nantong area. After standard curing, unconfined compressive strength tests were carried out, and a sample database containing multidimensional features, such as soil parameters, mix ratios, and ages, was constructed. The sparrow search algorithm (SSA) [25,26] was introduced into the parameter optimization process of the BP neural network. By simulating the foraging behavior of sparrow populations, global optimization of the initial weights and thresholds was achieved, and an intelligent prediction model for the strength of SSA-BP cement-solidified soil was established. To verify the superiority of the model, a four-dimensional comparison system was designed: a comprehensive comparison of prediction accuracy, convergence speed, and robustness was conducted with the BP neural network model [27,28] optimized by the particle swarm optimization (PSO) algorithm, the BP neural network model optimized by the genetic algorithm (GA) [29,30], and the BP neural network model with double hidden layers, providing a precise and reliable strength prediction tool for regional cement soil projects.

## 2. SSA-BP Model

### 2.1. BP Neural Network

**Figure 1:**
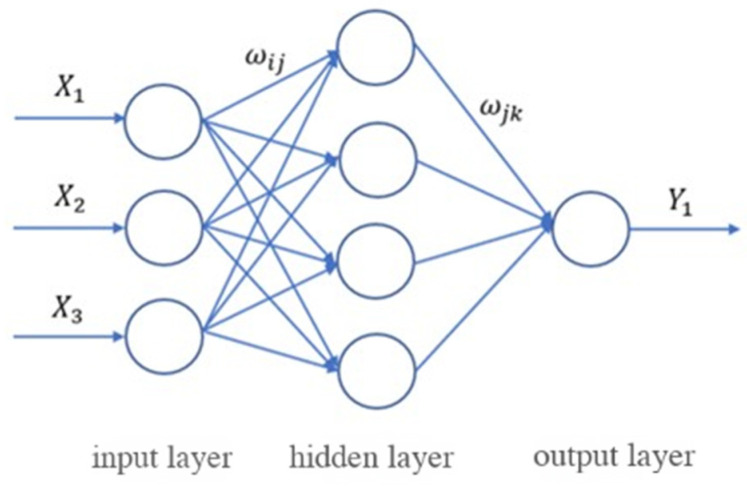
The graphic of the BP neural network with a 3-4-1 topology.

The BP neural network is a multi-layer feedforward network, and its core features are the signal forward propagation and error backpropagation mechanisms [31]. The typical neural network topology (3-4-1 Topology) consists of an input layer, a hidden layer, and an output layer (Figure 1). Data is transmitted layer by layer from the input layer through the hidden layer to the output layer, and the output values of each layer are processed by the activation function. The error between the network output and the expected value adjusts the weights and thresholds through backpropagation, so that the prediction result gradually approaches the real value.

The training process for the BP neural network is as follows:

(1) Parameter initialization: Determine the network structure (the number of neurons n in the input layer, the number of neurons m in the output layer, and the number of neurons l in the hidden layer) based on the sample features. Randomly initialize the weights $\omega_{ij}$ and $\omega_{jk}$ between the neurons of the input layer, the hidden layer, and the output layer, the threshold a of the hidden layer, and the threshold b of the output layer. Set the learning rate and the neuron activation function.
(2) Forward propagation calculation: Calculate the output $H_{j}$ of the j-th hidden layer based on the input value $X_{i}$ of the i-th input layer, the weight $\omega_{ij}$ between the input layer and the hidden layer, and the threshold $a_{j}$ of the hidden layer.
  (1) $$H_{j}=f(\sum_{i=1}^{n}\omega_{ij}x_{i}-a_{j})j=1,2\ldots ,l$$
  where f is the activation function of the hidden layer, and the tansig excitation function is adopted: $f(x)=2/[1+\exp\left(-2x\right)]-1$.

Calculate the output $O_{k}$ of the k-th output layer based on the output $H_{j}$ of the j-th hidden layer, the weights $\omega_{jk}$ between the hidden layer and the output layer, and the threshold $b_{k}$ of the output layer.

(2) $$O_{k}=f(\sum_{j=1}^{l}\omega_{jk}h_{j}-{ab}_{k})k=1,2\ldots ,m$$

where f is the activation function of the output layer, and the purelin activation function is adopted: f(x)=x.

(3) Error calculation and backpropagation: calculate the global mean squared error E based on the network’s predicted output $O_{k}$ and the expected output $y_{k}$.
  (3) $$E=\frac{1}{2m}\sum_{t=1}^{T}\sum_{k=1}^{m}{\left(o_{k}(t)-y_{k}(t)\right)}^{2}t=1,2,\ldots ,T$$

Based on the gradient descent method, use the global mean squared error *E* for backpropagation to update the network connection weights ω and b.

(4) $$∆\omega =-\eta \frac{\partial E}{\partial \omega };\;∆b=-\eta \frac{\partial E}{\partial b}$$

(4) Iteration termination determination: If the error *E* meets the preset precision or the maximum number of iterations is reached, terminate the training; otherwise, return to step (2) to continue the optimization.

### 2.2. Sparrow Search Algorithm (SSA)

The sparrow search algorithm is a new type of swarm intelligence optimization algorithm proposed in the past two years, which is mainly inspired by the foraging and anti-predation behaviors of sparrow groups [32,33]. In the population, discoverers are usually more likely to search for areas with abundant food; that is, they have good fitness values. Followers will change according to the direction of the foraging area of the discoverers. The positions of discoverers and followers will change dynamically, but the ratio of discoverers to followers in the group remains the same. The anti-predation behavior of sparrows is added; that is, there will be a certain number of sentinels in the group to warn of approaching danger, thereby updating the population’s positions.

Here, the position of each sparrow serves as the fitness value. Discoverers with better fitness values will be retained and provide the search areas and directions for the remaining followers. The formula for updating the positions of discoverers is as follows:

(5) $$x_{i}^{t+1}=\left\{\begin{array}{c} x_{i}^{t}\cdot \exp\left(-\frac{1}{\alpha \cdot iter_{max}}\right)\;R_{2}<ST\\ \;\;x_{i}^{t}+Q\;{\;\;\;\;\;\;\;\;\;\;\;\;\;\;\;\;\;\;\;\;\;\;\;\;\;\;R}_{2}\geq ST\; \end{array}\right.$$

where t represents the current number of evolutions, $iter_{max}$ represents the maximum number of evolutions, $x_{i}^{t}$ represents the position of the *i*-th sparrow, α is a random number between 0 and 1, and *ST* represents the safety value. $R_{2}<ST$ means there are no predators around the search range at this time; that is, the discoverers can search normally. $R_{2}\geq ST$ means that the sparrows in the population have found a predator and issued a warning to the group, and all sparrows will change their positions.

Followers will follow the positions of discoverers during the foraging process. The followers who join later will fly in other directions to change their positions due to the lack of food. The formula for updating the positions of followers is as follows:

(6) $$X_{i}^{t+1}=\left\{\begin{array}{c} Q\cdot \exp\left(\frac{X_{worst}-X_{i}^{t}}{i^{2}}\right)\;\;\;\;\;\;\;\;\;\;\;i>\frac{n}{2}\\ X_{P}^{t+1}+\left|X_{i}^{t}-X_{P}^{t+1}\right|\cdot A^{T}{\left(AA^{T}\right)}^{-1}\; \end{array}\right.$$

where $X_{P}$ is the optimal position occupied by the current discoverers, $X_{worst}$ is the worst position in the current global scope, and A is a random value in {−1, 1}. Meanwhile, the identities of followers and discoverers will be updated after each iteration, and some followers will replace the positions of discoverers.

During the sparrow foraging process, some sparrows can act as sentinels to alert to dangers. If they sense danger, they quickly adjust their own positions. There are two cases. When the fitness value is higher than the optimal fitness value, it means they are at the edge of the population, so they move towards the middle of the population. When the fitness value is equal to the optimal fitness value, it means the sparrows in the middle position sense danger and need to get closer to other sparrows. The position update formula for sentinels is as follows:

(7) $$X_{i}^{t+1}=\left\{\begin{array}{c} X_{best}^{t}+\beta \cdot \left|X_{i}^{t}-X_{best}^{t}\right|\;\;f_{i}>f_{b}\\ X_{i}^{t}+K\cdot \frac{\left|X_{i}^{t}-X_{worst}^{t+1}\right|}{\left(f_{i}-f_{w}\right)}\;f_{i}\leq f_{b}\; \end{array}\right.$$

where β is a random number following the normal distribution [0, 1], and $K\in \left[-1,\;1\right]$ is a random number. $f_{b}$ is the best fitness value, $f_{w}$ is the worst fitness value, and $f_{i}$ is the current individual fitness value.

### 2.3. Optimization of BP Neural Network Model Based on Sparrow Search Algorithm

**Figure 2:**
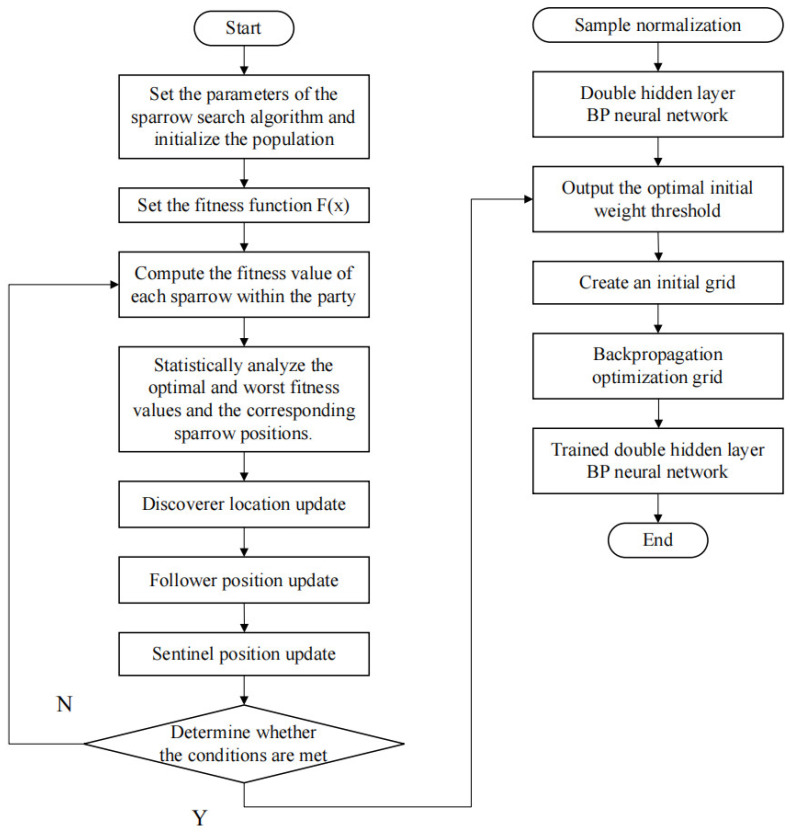
Flowchart of the SSA-BP neural network model.

This paper proposes an SSA-BP model that optimizes the initial weights and thresholds of a double-hidden-layer BP neural network based on the SSA sparrow search algorithm. This model can solve the problems of network non-convergence or getting stuck in local optimal values caused by manual parameter selection, thereby improving the accuracy and generalization ability of the BP neural network. The specific process is shown in Figure 2.

The algorithm initializes SSA parameters, generates the initial population, and defines the fitness function to evaluate individual fitness F(x) while recording optimal/worst solution positions. It then iteratively updates the discoverers’ exploratory positions, followers’ approaching movements, and scouts’ random escapes until termination conditions are met; subsequently, it normalizes sample data, initializes a dual-hidden-layer BP neural network with SSA-optimized weights/thresholds, and finally refines the network through backpropagation to output the trained prediction model.

## 3. SSA-BP Strength Prediction Model for Mineral Powder Cement-Solidified Soil

### 3.1. Experimental Sample

This study conducted indoor cement–soil mix proportion tests based on the “Standard for Geotechnical Tests”. The test soil samples are taken from typical sandy soil (silt) and clay (silty clay interbedded with silt) in the Nantong area. The basic physical and mechanical indicators are shown in Table 1.

Before the test, the soil samples were pre-treated by air-drying, crushing, and screening out impurities. Based on five factors and four levels, including cement content (12%, 16%, 20%, 24%), mineral powder content (0%, 4%, 8%, 12%), bentonite content (0%, 5%, 10%, 15%), water–cement ratio (1.3, 1.5, 1.8, 2.0), and curing age (7 d, 14 d, 28 d, 45 d), 32 groups of orthogonal test schemes were designed [34,35]. Six parallel cubic specimens of 70.7 mm × 70.7 mm × 70.7 mm were prepared for each group, and a total of 192 specimens were formed. The test schemes are shown in Table 2. In the table, SC represents the sand–mineral powder–cement solidified soil; NC represents the cohesive soil–mineral powder–cement solidified soil.

**Figure 3:**
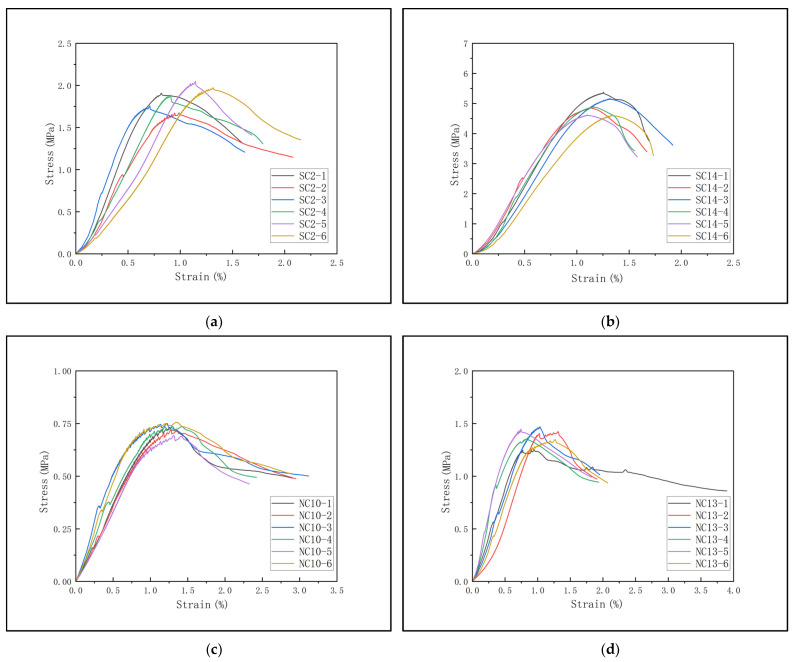
Stress–strain curves of some specimens. (**a**) Sample set of sandy soil, mineral powder, and cement-solidified soil 1; (**b**) sample set of sandy soil, mineral powder, and cement-solidified soil 2; (**c**) sample group of clay, mineral powder, and cement-solidified soil 1; (**d**) sample group of clay, mineral powder, and cement-solidified soil 2.

The specimens are placed in a standard curing chamber with a temperature of 20 ± 2 °C and a relative humidity of ≥95% for curing until the specified age is reached. After drying the surface, their volume and mass are measured. The CMT5504 universal testing machine is used to conduct the unconfined compressive strength test at a loading rate of 0.05 kN/s. After eliminating the abnormal data with a dispersion degree exceeding 15%, the average value is taken as the final strength value (Table 2). Figure 3 shows some of the test result curves. The stress–strain curves obtained from the tests exhibit typical characteristics of elastic deformation and plastic failure, with small data dispersion, indicating that the specimens have good uniformity and the test results are reliable.

### 3.2. Sample Data Processing

As shown in Table 3, the model selects five parameters—cement content, slag powder content, bentonite content, comprehensive moisture content, and age—as the input layer variables, and takes the unconfined compressive strength of cement soil as the output layer variable. Among them, the cement content, slag powder content, bentonite content, and age are directly input according to the mix ratio, while the comprehensive moisture content is calculated by the water–cement ratio according to Equation (8):

(8) $$\omega_{All}=\omega_{0}+\frac{w}{c}*\left(1+\omega_{0}\right)*(\alpha +\beta )$$

In the formula, $\omega_{All}$ is the comprehensive moisture content, $\omega_{0}$ is the initial natural moisture content, w/c is the water–cement ratio, α is the cement content, and *β* is the mineral powder content. In the experimental dataset, the sand–mineral powder–cement-solidified soil model uses 84 sets of mix proportions and their corresponding age compressive strengths as the sample set, while the clay model uses 93 sets of sample data. All data are normalized and then input into the neural network for training and testing.

The optimal selection of the number of hidden layer nodes directly affects the prediction performance of the neural network. In this paper, a method combining preliminary screening using empirical formulas and comparative verification of MSE is adopted to determine the optimal structure. The theoretical interval of the number of hidden layer nodes is determined by Equation (9):

(9) $$l=\sqrt{\left(m+n\right)}+a$$

In the formula, m is the number of nodes in the output layer, n is the number of nodes in the input layer, a is a constant between 0 and 10, and l is the number of nodes in the hidden layer. Thus, the number of nodes in the hidden layer was determined to be approximately between 3 and 12. The mean squared error (MSE) of models with different hidden layer node counts (within the 3–12 range) was calculated using 12 groups of test samples. The training accuracy under different numbers of hidden nodes was compared based on these MSE values, where a smaller mean squared error indicates higher accuracy. The comparison of BP neural network models with different numbers of neurons in the hidden layer is shown in Table 4 and Table 5.

**Figure 4:**
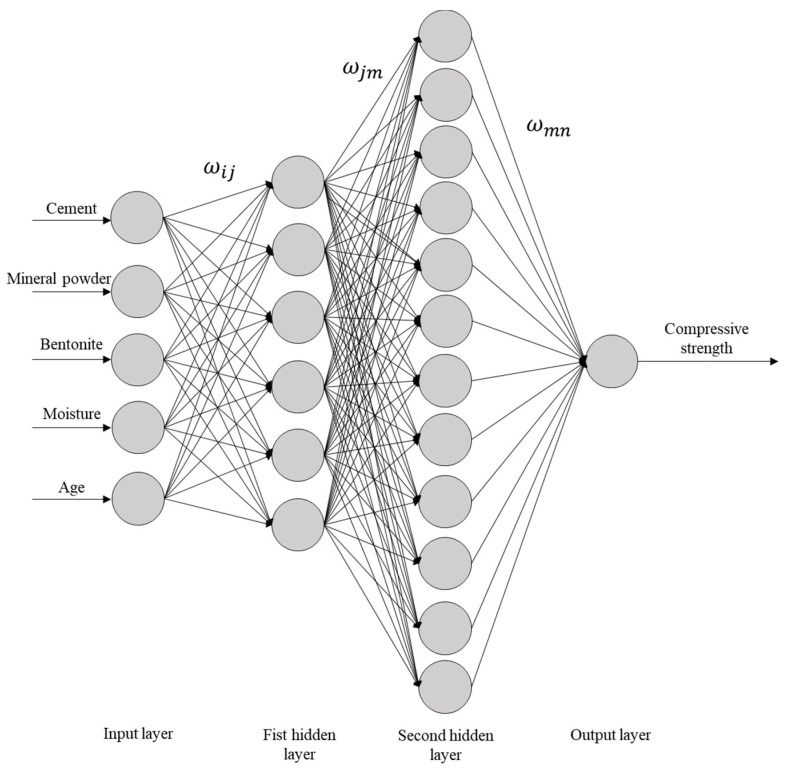
Structural diagram of a neural network for cement-solidified soil with sand and mineral powder.

**Figure 5:**
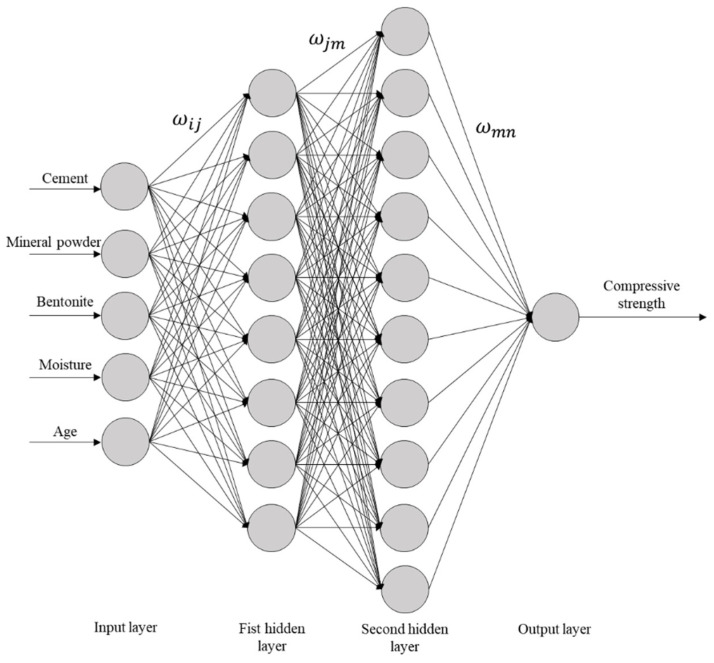
Structural diagram of a neural network for cement-solidified soil with clay and mineral powder.

Comparing the MSE values under different numbers of nodes showed that the sand model reaches its optimal state when there are eight nodes in the first hidden layer and 10 nodes in the second hidden layer, with a 5-8-10-1 topological structure. The total number of weight thresholds is 149 (including 40 input-hidden layer weights, 80 inter-hidden layer weights, and 10 output weights); the clay model achieves the minimum value when there are 6 nodes in the first hidden layer and 12 nodes in the second hidden layer, with a 5-6-12-1 network architecture. The total number of weight thresholds is 133 (including 30 input-hidden layer weights, 72 inter-hidden layer weights, and 12 output weights). The specific network connection topologies are shown in Figure 4 and Figure 5. The structural differences confirm the soil-dependent characteristics of the solidification mechanism of marine sedimentary soils.

### 3.3. Model Training and Result Analysis

SSA-BP strength prediction models for sand and clay mineral powder cement-solidified soil were constructed on the MATLAB 2018a platform. The sand model uses 84 sets of data (72 sets for the training set and 12 sets for the test set), and the clay model uses 93 sets of data (81 sets for the training set and 12 sets for the test set). The model uses the comprehensive error of the training set as the fitness function (Equation (10)) and optimizes the weights and thresholds of the neural network through SSA.

(10) $$error=\sum_{i=1}^{j}\left|Predictedvalue-Measuredvalue\right|$$

where *j* represents the number of samples in the training set. For the sandy soil model, *j* is 72, and for the clay soil model, *j* is 81. The algorithm initializes and generates multiple sparrow individuals (i.e., combinations of weight and threshold). During the iteration process, the positions of the individuals with the lowest fitness are retained. After optimization for a preset number of rounds, the optimal weight and threshold parameters are obtained.

**Figure 6:**
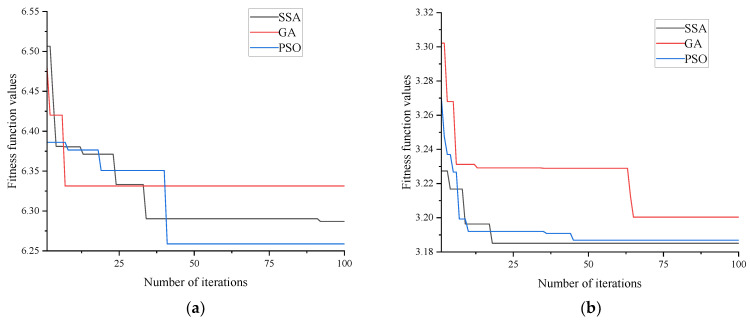
Comparison of fitness function values for the SSA, the GA, and the PSO algorithm in two soil models. (**a**) Sand, sandy soil, ore powder, and cement–soil model; (**b**) clay–mineral powder–cement–soil model.

Figure 6 compares the fitness curves of the SSA, the GA, and the PSO algorithm in two soil models. The results show that the fitness value of the GA is significantly higher than those of the SSA and PSO, and it is prone to falling into local optima. The SSA has the fastest convergence speed in the double-hidden-layer BP network and can reach the global optimum more quickly. It is worth noting that the differences in the optimal fitness values of the three algorithms in the clay model are relatively small, indicating that the strength prediction model of clay solidified soil has higher stability.

**Figure 7:**
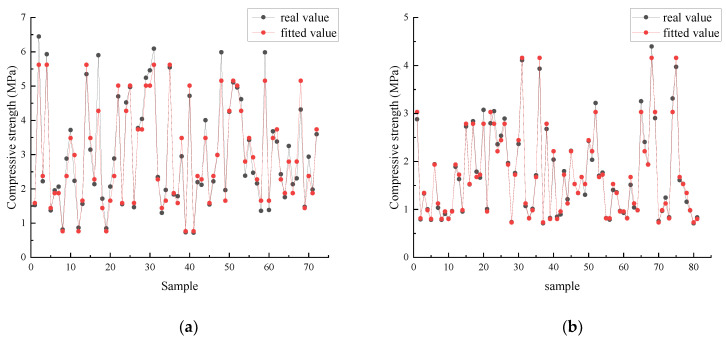
Comparison of actual vs. fitted values in the training set for two soil–cement–mineral powder models. (**a**) Sand, sandy soil, ore powder, and cement–soil model; (**b**) clay–mineral powder–cement–soil model.

Further, an SSA-BP model is established based on the optimal weight threshold, and the root mean square error (RMSE), mean relative error (MRE), and mean absolute error (MAE) are used to evaluate the prediction performance. Figure 7 shows that the fitted values of the model are highly consistent with the measured values. The MAEs of the training sets for sandy soil and clay are 0.28 and 0.07, respectively; the MREs are 9.36% and 4.00%, respectively, and the RMSEs are 0.38 and 0.11, respectively. The results of the test set indicate that the model can effectively predict the nonlinear relationship between the strength of mineral powder cement solidified soil and multiple factors, and has good engineering applicability.

### 3.4. Prediction and Evaluation of SSA-BP Neural Network Model

The strength of 12 groups of test samples was predicted based on the trained SSA-BP model. The results are shown in Table 6. The prediction performance indicators of the sandy soil, mineral powder, and cement-solidified soil model are as follows: MRE = 8.45%, MAE = 0.30 MPa, RMSE = 0.44 MPa, and R^2^ = 0.945. The corresponding indicators of the clay model are MRE = 6.63%, MAE = 0.15 MPa, RMSE = 0.20 MPa, and R^2^ = 0.992. Among them, the MRE of the test sets of the sandy soil and clay models increased slightly compared with that of the training sets, but the deviation range was small, indicating that the model did not show a significant overfitting phenomenon while maintaining high coupling.

In terms of prediction accuracy, the prediction error of sand strength is 0.30 MPa, and the error of clay is further reduced to 0.15 MPa. According to the analysis of RMSE and R^2^ indicators, the RMSE of the clay model is significantly lower than that of the sand model, and its R^2^ value is closer to the theoretical optimal value of 1, indicating that the strength prediction results of clay solidified soil have higher stability. The test results verify the strong generalization ability of the SSA-BP model under multi-soil conditions. Its error level meets the allowable error requirements (≤10%) for the strength detection of solidified soil in the “Technical Code for Ground Treatment of Buildings” (JGJ 79-2012), which can provide reliable predictions for engineering practice.

## 4. Comparative Analysis of SSA-BP and Other Models

To verify the effectiveness of the SSA in optimizing and selecting the initial weights and threshold parameters of the double-hidden-layer BP neural network, this study sets up a control experiment: the particle swarm optimization (PSO) and genetic algorithm (GA) are respectively used to optimize the weights and thresholds of the double-hidden-layer BP neural network, and the results are compared with the unoptimized original BP model. The parameter settings of each algorithm are shown in Table 7. Among them, SSA, PSO, and GA all use the same population size (N = 20) and number of iterations (T = 100) to ensure the fairness of the comparison.

**Figure 8:**
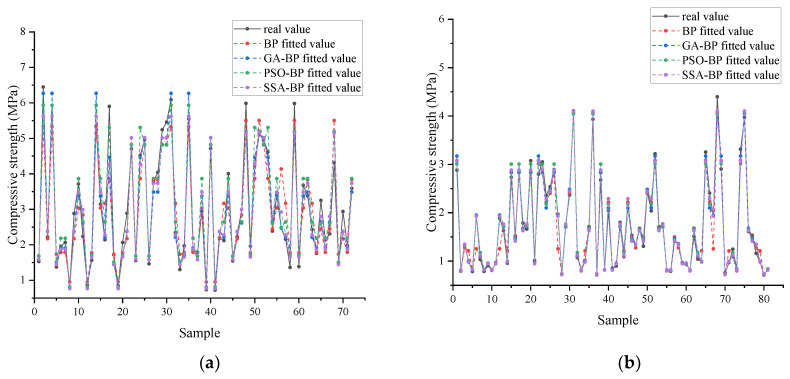
Comparison of different prediction models for cement-stabilized soils in the training set. (**a**) Sand, soil, ore powder, and cement–soil model; (**b**) clay–mineral powder–cement–soil model.

**Figure 9:**
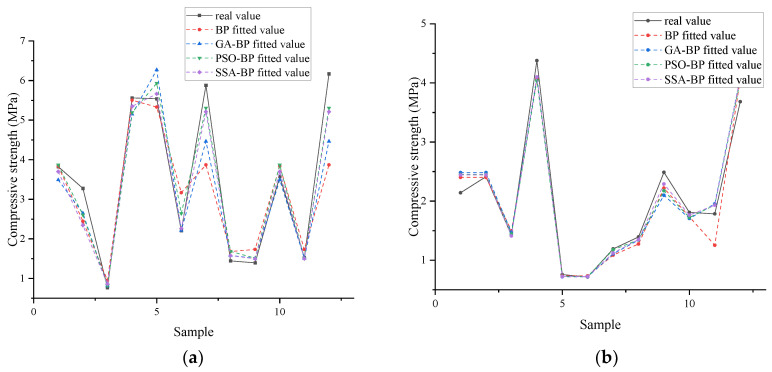
Comparison of different prediction models for mineral powder–cement-stabilized soils in the test set. (**a**) Sand, soil, ore powder, and cement–soil model; (**b**) clay–mineral powder–cement–soil model.

As can be seen in Figure 8 and Figure 9, all four fitting methods have good fitting accuracy and perform well on the test set. However, on the test set, the BP neural network has a relatively large error, indicating obvious overfitting. This shows that optimizing the initial weights and thresholds through optimization algorithms can significantly improve the accuracy of the BP neural network. When comparing the test sets, it is found that the prediction accuracy of PSO-BP is similar to that of SSA-BP, while the prediction accuracy of GA-BP is relatively low.

Based on the comparative analysis of the cement-solidified soil models of sandy soil and clayey soil with mineral powder (Table 8 and Table 9), the SSA-BP neural network shows significant advantages in both prediction accuracy and generalization ability. For the sandy soil model, the MAE of the test set of SSA-BP is 53.4% lower than that of the original BP model, and the RMSE is optimized by 56.3%. The determination coefficient of its test set is exactly the same as that of the training set, completely eliminating the serious overfitting phenomenon existing in the BP model. Compared with other optimization algorithms, the test set error of SSA-BP is 4.3% and 40.7% lower than that of PSO-BP and GA-BP, respectively. In the clay model, SSA-BP leads with the lowest test error, which is 6.1% and 15.1% lower than those of PSO-BP and GA-BP, respectively, and its MRE is 5.57%. Experiments show that by dynamically balancing the search path of the parameter space, the SSA reduces the error transfer rate from the training set to the test set by 38–65% compared with traditional optimization strategies under the same number of iterations, verifying its engineering robustness in the strength prediction of complex soil.

## 5. Analysis of Input Feature Weights of the Strength Prediction Model for Cement-Solidified Soil with Mineral Powder

Based on the analysis of the weights and thresholds of the double-hidden-layer BP neural network optimized by the SSA, this paper uses the weight contribution rate analysis method to establish a quantitative relationship between input variables and output indicators. This method calculates the relative contribution of each input feature in the prediction process by analyzing the weight matrix obtained after neural network training. First, the weight contribution rate $w_{im}$ of input feature i to each node m in the second layer is calculated by Equation (11).

(11) $$w_{im}=\sum_{j=1}^{8}{w1}_{ij}*{w2}_{jm}\left(\frac{{w1}_{ij}}{\sum_{i=1}^{5}{w1}_{ij}}\right)$$

In the formula, ${w1}_{ij}$ is the connection weight between the input node *i* and the *j*-th node in the first hidden layer, and ${w2}_{jm}$ is the connection weight between the j-th node in the first hidden layer and the m-th node in the second hidden layer. Secondly, the weight contribution rate $w_{i}$ of the input features to the output layer is transmitted layer by layer through Formula (12).

(12) $$w_{i}=\sum_{m=1}^{10}{w2}_{im}\;*\;{w3}_{mn}\left(\frac{{w2}_{im}}{\sum_{i=1}^{5}{w2}_{im}}\right)$$

where ${w3}_{mn}$ is the connection weight between the second hidden layer *m* and the output layer *n*. Finally, the contribution rate of each feature weight is obtained through the normalization process outlined in Equation (13).

(13) $$P_{i}=w_{i}/\sum_{i=1}^{5}w_{i}$$

This method effectively deconstructs the “black box” characteristic of neural networks, and its calculation results are shown in Table 10.

There are significant differences in the strength influence mechanisms of the input features of cement-solidified soil made of sand and clay with mineral powder. For the sand model, the curing age becomes the dominant factor with a weight contribution rate of 73.19%. Its contribution value is 5.6 times that of the mineral powder content and 9.9 times that of the cement content, which is consistent with the conclusion that the range value of the age is the largest in the orthogonal test. However, the model contribution rate is 69.5% higher than the test value. It is speculated that this is due to the enhanced learning of the time-series characteristics, which is caused by the fact that the data from the 7-day curing age accounts for 62% in the training set samples. The contribution rate advantage of the mineral powder content confirms the key role of the mineral powder in the cementation of sand particles.

In the clay model, the contribution rate of the mineral powder content ranks first, followed by the curing age and the comprehensive moisture content. Among them, the contribution rate of the moisture content exceeds that of the cement content, which can be reasonably explained by the sensitivity mechanism of the interaction between clay particles and water. The feature weight rankings of the two types of models are highly consistent with the conclusions of the orthogonal test.

## 6. Conclusions

Based on the research on the compressive strength prediction model of cement-solidified soil made from Nantong marine sedimentary sand and clay mineral powder, through the comparative analysis of SSA-BP, PSO-BP, GA-BP, and the original BP models, the following conclusions are drawn:

(1) Although the original BP neural network has strong sample fitting ability, the randomness of its initial weights and thresholds leads to severe overfitting in the sand test set and significant prediction errors. After optimization by the SSA, PSO algorithm, and GA, the RMSE of the sand test set is reduced by up to 56.3%, and the MAE of the clay test set is decreased by 27.5%, which confirms that the intelligent algorithms can effectively improve the stability and generalization ability of the model.

(2) The SSA-BP model shows the best prediction accuracy in both types of soil: the MRE of the sand test set is 10.89% lower than that of the BP model, and the MRE of the clay test set is 3.00% lower than that of the BP model. In the sand model, the SSA-BP algorithm improves the R^2^ of the test set to 0.990 through a dynamic search strategy, which is the same as that of the training set, completely eliminating overfitting; in the clay model, the RMSE of the SSA-BP model is lowest, being 6.1% and 15.1% lower than the PSO-BP and GA-BP models, respectively.

(3) The order of prediction accuracy for sand is SSA-BP > PSO-BP > GA-BP > BP. The R^2^ difference of the test set of the GA-BP model reaches 0.134 due to premature convergence, indicating its local optimum defect; in the clay model, although none of the four algorithms shows overfitting, the SSA-BP model is significantly better than the PSO-BP and GA-BP models. The RMSE of the SSA in sand and clay prediction is 4.3% and 6.1% lower than that of the PSO algorithm, respectively, demonstrating the advantage of its global search ability.

(4) The analysis of weight contribution rate reveals that the dominant factor for the strength of sandy soil is the curing age, and its contribution value is 5.6 times that of the mineral powder content, reflecting the time-effect control of the age on the hydration process of sandy soil. For the strength of clay, the mineral powder content is the core, and the interaction between the mineral powder and clay particles is significant. The ranking of the factors affecting the strength of the two types of soil is highly consistent with the conclusion of the orthogonal test, indicating that the SSA-BP model has both prediction accuracy and mechanism interpretability.

## Figures and Tables

**Table 1:** Basic physical and mechanical indexes of soil samples.

Natural Moisture Content/%	Volumetric Weight/(kN·m^−3^)	Void Ratio	Liquidity Index	Coefficient of Non-Uniformity	Soil Classification
33.6	18.1	0.968	0.94	7.18	Silty clay interbedded with silt
24.2	19.5	0.681	\	4.31	Silt

**Table 2:** Orthogonal test scheme and test results of sand and clay soil–cement.

ID	Cement Content	Mineral Powder Content	Bentonite	Water–Cement Ratio	Age (d)	Compressive Strength (MPa) SC/NC
SC1/NC1	1 (12%)	1 (0%)	1 (0%)	1 (1.3%)	1 (7 d)	0.792/0.814
SC2/NC2	1 (12%)	2 (4%)	2 (5%)	2 (1.5%)	2 (14 d)	1.956/1.317
SC3/NC3	1 (12%)	3 (8%)	3 (10%)	3 (1.8%)	3 (28 d)	2.855/1.667
SC4/NC4	1 (12%)	4 (12%)	4 (15%)	4 (2.0%)	4 (45 d)	3.699/2.258
SC5/NC5	2 (16%)	1 (0%)	2 (5%)	3 (1.8%)	4 (45 d)	1.720/0.947
SC6/NC6	2 (16%)	2 (4%)	1 (0%)	4 (2.0%)	3 (28 d)	2.558/1.133
SC7/NC7	2 (16%)	3 (8%)	4 (15%)	1 (1.3%)	2 (14 d)	3.488/2.377
SC8/NC8	2 (16%)	4 (12%)	3 (10%)	2 (1.5%)	1 (7 d)	2.233/1.738
SC9/NC9	3 (20%)	1 (0%)	3 (10%)	4 (2.0%)	2 (14 d)	1.464/0.732
SC10/NC10	3 (20%)	2 (4%)	4 (15%)	3 (1.8%)	1 (7 d)	1.554/0.813
SC11/NC11	3 (20%)	3 (8%)	1 (0%)	2 (1.5%)	4 (45 d)	5.224/3.061
SC12/NC12	3 (20%)	4 (12%)	2 (5%)	1 (1.3%)	3 (28 d)	5.817/4.079
SC13/NC13	4 (24%)	1 (0%)	4 (15%)	2 (1.5%)	3 (28 d)	2.613/1.454
SC14/NC14	4 (24%)	2 (4%)	3 (10%)	1 (1.3%)	4 (45 d)	5.009/2.878
SC15/NC15	4 (24%)	3 (8%)	2 (5%)	4 (2.0%)	1 (7 d)	2.471/0.814
SC16/NC16	4 (24%)	4 (12%)	1 (0%)	3 (1.8%)	2 (14 d)	5.392/1.317

**Table 3:** Input and output parameter ranges of the neural network.

	Parameter	Sandy Soil		Clay	
		Minimum	Maximum	Minimum	Maximum
Model input	Cement content (%)	12	24	12	24
	Mineral powder content (%)	0	12	0	12
	Bentonite content (kg/m^3^)	0	15	0	15
	Comprehensive moisture content (%)	43.58	104.68	54.44	120.17
	Age (d)	7	45	7	45
Model output	Compressive strength (MPa)	0.72	6.45	0.71	4.40

**Table 4:** Hidden layer neuron numbers vs. MSE for sand–mineral powder–cement-stabilized soil prediction model.

Number of Hidden Layer Nodes	3	4	5	6	7	8	9	10	11	12
3	0.396	0.183	0.497	0.362	0.500	0.293	1.808	0.596	0.466	0.307
4	0.708	0.201	0.435	0.523	0.323	0.345	0.323	0.219	0.336	0.259
5	0.316	0.414	0.308	0.180	0.294	0.316	0.225	0.251	0.191	0.423
6	0.303	0.247	0.580	0.502	0.252	0.313	0.315	0.264	0.287	0.246
7	0.264	0.302	0.807	0.286	0.224	0.225	0.386	0.331	0.482	0.259
8	0.407	0.504	0.388	0.334	0.475	0.282	0.276	0.137	0.261	0.261
9	0.191	0.625	0.552	0.467	0.266	0.430	0.641	0.233	0.270	0.787
10	0.478	0.480	0.257	0.362	0.256	0.205	0.403	0.292	0.343	0.295
11	0.281	1.039	0.436	0.142	0.334	0.288	0.270	0.237	0.275	0.267
12	1.131	0.659	0.285	0.186	0.318	0.302	56.687	0.257	0.237	0.466

**Table 5:** Hidden layer neuron numbers vs. MSE for clay–mineral powder–cement-stabilized soil prediction model.

Number of Hidden Layer Nodes	3	4	5	6	7	8	9	10	11	12
3	0.052	0.044	0.062	0.043	0.309	0.043	0.059	0.043	0.041	0.039
4	0.041	0.045	0.042	0.039	0.045	0.037	0.038	0.039	0.037	0.034
5	0.041	0.034	0.040	0.037	0.044	0.035	0.045	0.050	0.049	0.040
6	0.246	0.040	0.041	0.037	1.055	0.047	0.037	0.036	0.038	0.031
7	0.038	0.050	0.038	0.041	0.044	0.041	0.042	0.045	0.042	0.041
8	0.043	0.058	0.037	0.525	0.038	0.041	0.042	0.047	0.045	0.040
9	0.039	0.042	0.042	0.040	0.035	0.043	0.037	0.040	0.040	0.042
10	0.040	0.042	0.034	0.036	0.037	0.038	0.035	0.051	0.045	0.038
11	0.034	0.040	0.492	0.042	0.039	2.138	0.045	0.039	0.045	0.037
12	0.039	0.043	0.039	0.037	0.046	0.038	0.863	0.041	0.045	0.044

**Table 6:** Test set evaluation metrics of the SSA-BP model.

Evaluation Indicators	SSA-BP Sand–Cement Soil Model	SSA-BP Clay–Cement Soil Model
MRE	8.45%	6.63%
MAE	0.30	0.15
RMSE	0.44	0.20
Coefficient of determination (R^2^)	0.945	0.992

**Table 7:** Core parameters of three optimization algorithms and BP neural network configuration.

SSA Parameters		GA Parameters		PSO Algorithm Parameters		BP Neural Network Parameters	
Number of iterations	100	Number of evolutions	100	Number of iterations	100	Maximum number of training times	200
Population size	20	Population size	20	Population size	20	Learning rate	0.1
Discoverer ratio	0.7	Crossover probability	0.2	Acceleration factor	1.49445	Learning objectives	0.01
Watcher ratio	0.1	Mutation probability	0.05				
Alert threshold	0.6						

**Table 8:** Comparative statistics of computational results by different prediction models in sand–mineral powder–cement-stabilized soil.

Serial Number	Model	Training Set				Test Set			
		MAE	MRE	RMSE	R^2^	MAE	MRE	RMSE	R^2^
1	BP NN	0.419	15.45%	0.574	0.978	0.635	19.34%	0.972	0.737
2	GA-BP NN	0.270	9.03%	0.375	0.991	0.470	10.70%	0.717	0.857
3	PSO-BP NN	0.322	11.41%	0.406	0.989	0.339	9.98%	0.444	0.950
4	SSA-BP NN	0.276	9.36%	0.385	0.990	0.296	8.45%	0.425	0.990

**Table 9:** Comparative statistics of computational results by different prediction models in clay–mineral powder–cement-stabilized soil.

Serial Number	Model	Training Set				Test Set			
		MAE	MRE	RMSE	R^2^	MAE	MRE	RMSE	R^2^
1	BP NN	0.122	7.30%	0.206	0.993	0.182	8.57%	0.242	0.948
2	GA-BP NN	0.075	4.04%	0.118	0.998	0.166	7.23%	0.219	0.957
3	PSO-BP NN	0.078	4.23%	0.118	0.998	0.142	5.86%	0.198	0.965
4	SSA-BP NN	0.075	4.08%	0.113	0.998	0.132	5.57%	0.186	0.969

**Table 10:** Weight-based contribution rates of input features in the prediction model.

Influencing Factors	Weight of Solidified Sandy Soil, Ore Powder, and Cement		Weight of Clay, Ore Powder, and Cement-Solidified Soil	
	Weight Contribution	Weight Contribution Rate	Weight Contribution	Weight Contribution Rate
Cement content	26.01	7.39%	4.22	9.53%
Mineral powder content	46.23	13.13%	15.80	35.69%
Bentonite content	5.87	1.67%	0.94	2.13%
Comprehensive moisture content	16.31	4.63%	10.28	23.23%
Age	257.71	73.19%	13.02	29.42%

## Data Availability

The raw data supporting the conclusions of this article will be made available by the authors on request.
